# Potential Application of Plant-Based Functional Foods in the Development of Immune Boosters

**DOI:** 10.3389/fphar.2021.637782

**Published:** 2021-04-20

**Authors:** Linlin Jiang, Guoqing Zhang, Ye Li, Guirong Shi, Minhui Li

**Affiliations:** ^1^ Department of Pharmacy, Inner Mongolia Medical University, Hohhot, China; ^2^ Inner Mongolia Hospital of Traditional Chinese Medicine, Hohhot, China; ^3^ Baotou Centre Hospital, Baotou, China; ^4^ Pharmaceutical Laboratory, Inner Mongolia Institute of Traditional Chinese Medicine, Hohhot, China; ^5^ Inner Mongolia Key Laboratory of Characteristic Geoherbs Resources Protection and Utilization, Baotou Medical College, Baotou, China

**Keywords:** immune system, plant-based functional food, immune booster, disease treatment, bioactive compound

## Abstract

Immune dysfunction, which is responsible for the development of human diseases including cancer, is caused by a variety of factors. Therefore, regulation of the factors influencing the immune response is a potentially effective strategy to counter diseases. Presently, several immune adjuvants are used in clinical practice to enhance the immune response and host defense ability; however, synthetic drugs can exert negative side effects. Thus, the search for natural products of plant origin as new leads for the development of potent and safe immune boosters is gaining considerable research interest. Plant-based functional foods have been shown to exert several immunomodulatory effects in humans; therefore, the application of new agents to enhance immunological and specific host defenses is a promising approach. In this comprehensive review, we have provided an up-to-date report on the use as well as the known and potential mechanisms of bioactive compounds obtained from plant-based functional foods as natural immune boosters. Plant-based bioactive compounds promote immunity through multiple mechanisms, including influencing the immune organs, cellular immunity, humoral immunity, nonspecific immunity, and immune-related signal transduction pathways. Enhancement of the immune response in a natural manner represents an excellent prospect for disease prevention and treatment and is worthy of further research and development using approaches of modern science and technology.

## Introduction

**FIGURE 1:**
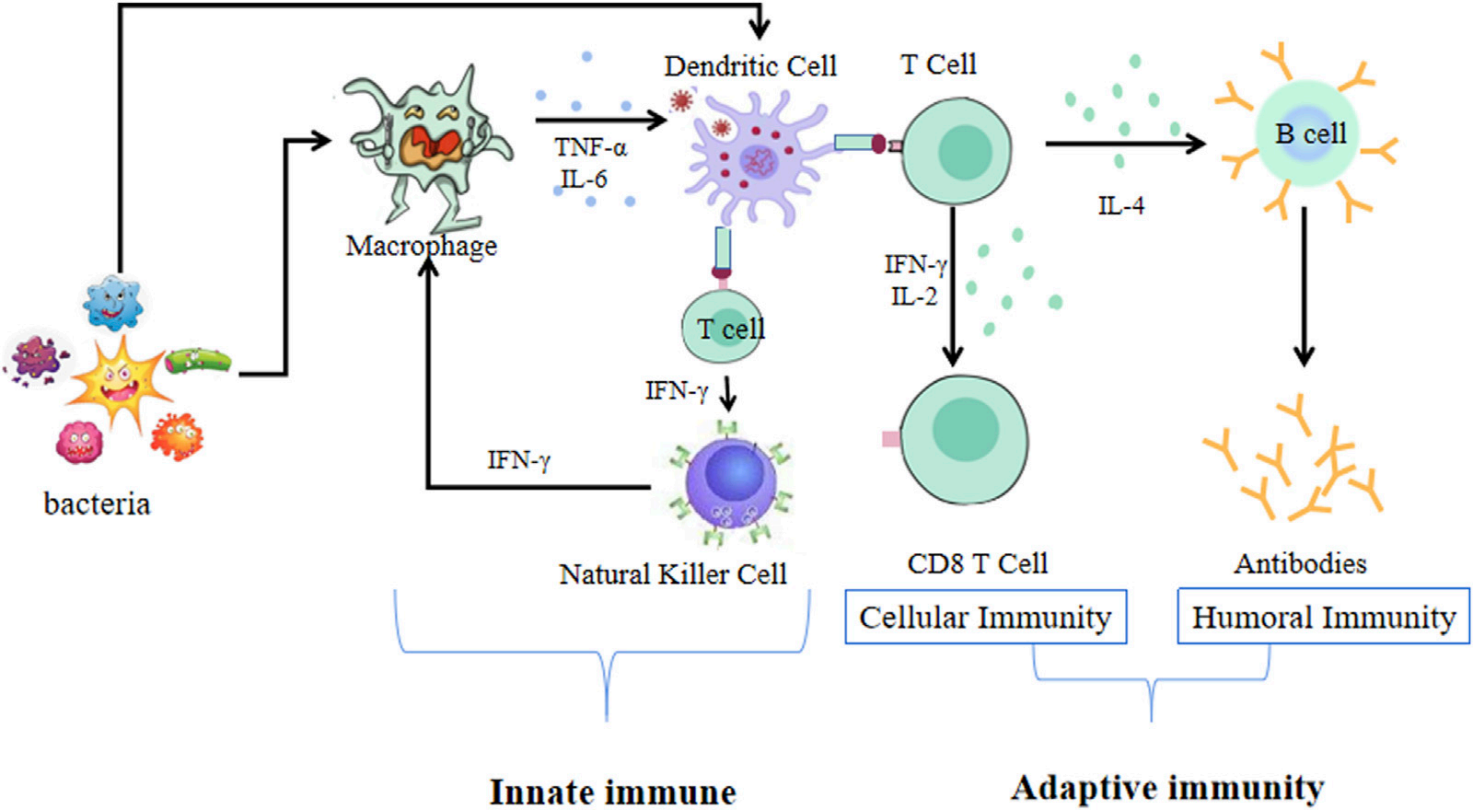
The immune system of innate and adaptive immunity.

The immune system is one of the most complex biological systems in the body. It is a multifaceted and sophisticated network of specialized organs, cells, proteins, and chemicals, and plays an essential role in conferring protection against various pathogens (such as bacteria, viruses, and fungi), and cancer cells (Carr and Maggini, 2017). It is well known that host immunity is constituted by innate (non-specific) and adaptive (specific) immunity (Figure 1) (Orlowsky and Kraus, 2015; Nicholson, 2016). Immune system dysfunction renders an organism sensitive to pathogens, which can lead to the development of diseases, such as allergic diseases, rheumatoid arthritis, and inflammatory bowel diseases (Williams et al., 2017b; Ding et al., 2018; García et al., 2020). Since immunity is fundamental to the health of the host and plays an important role in preventing diseases, increased research efforts have been engaged at improving immune function. Currently, the clinical application of immunomodulators mainly includes immune adjuvants, such as aluminum hydroxide, Freund’s adjuvant, and albumen adjuvants (Yu et al., 2016; Cronkite and Strutt, 2018). However, these adjuvants have been reported to cause local stimulation, tissue damage, and carcinogenesis. Additionally, neither aluminum hydroxide nor Freund’s adjuvant can induce a strong cellular immune response (Chen et al., 2019). Although albumen adjuvants have recently been used as the primary treatment strategy for improving the immune function of individuals, their application is limited by their high costs and side effects (Chen and Zhan, 2019). It is therefore necessary to explore natural, safer, and more effective adjuvants that can enhance immune systems function by activating immune cells and by modulating immune molecules.

Plant-based functional foods are derived from natural or processed plant foods that contain known or unknown bioactive components (Kumar et al., 2018). In recent years, they have been extensively consumed because of their high bioactive components and health benefits (Mohamad et al., 2020). Notably, growing evidence from pre-clinical research has shown that plant-based functional foods can reduce the risk of developing various disorders, such as diabetes, cancer, cardiovascular disease, hyperlipidemia, and hyperuricemia (Andrea et al., 2017; Gong et al., 2019; Mehmood et al., 2019; Gong et al., 2020; Jiang et al., 2020). Owing to a growing awareness of the capabilities of plant-based functional foods to combat diseases, studies on immune boosters based on plant-based functional foods have received substantial attention, and an increased number of individuals continue to choose plant-based functional foods to improve immune system functions (Davoodvandi et al., 2019; Shafabakhsh et al., 2019). For example, previous studies have reported that active polysaccharides obtained from *Ganoderma* may evoke an immune defense response against tumor growth, viruses, bacteria, and fungal pathogens via modulation of lymphocytes and myeloid cells, thereby indicating a potential application for the modulation of the host immune system (Ren et al., 2020). Furthermore, astragalus, ginseng, and *Op* *hiocordyceps sinensis* have also been reported to exhibit appreciable immune enhancement effects (Chen and Zhan, 2019; Huang et al., 2019; Kim et al., 2019; Chen Z. et al., 2020). In general, the active ingredients of plant functional foods that enhance immunity mainly include polysaccharides, saponins, flavonoids, alkaloids. This review highlights the biological components of plant-based functional foods with immune-boosting effects and their utility in immune-enhancing applications, including those in the development of new treatment strategies for diseases.

## Bioactive Components of Plant-Based Functional Foods and Their Immune-Boosting Effects

### Polysaccharides

Polysaccharides are a class of natural macromolecules consisting of glycosidically linked carbohydrate monomers (Ferreira et al., 2015). Polysaccharides obtained from natural sources are known to exhibit various biological activities, includg immune regulatory, anti-tumor, and anti-inflammatory activities (Liu M. et al., 2018; Meng et al., 2018). Indeed, the immune-enhancing effects of polysaccharides have garnered considerable attention in recent years because of their low toxicity and few side effects (Huang et al., 2020). Recently, polysaccharides have been shown to possess immune-boosting effects *in vitro* and *in vivo* as evidenced by the promotion of immune organ development and the secretion of immune-related molecules. Furthermore, while polysaccharides can promote the activation of the antigen-specific immune system, they can also enhance the innate immune functions of the body, which renders them an ideal potential adjuvant (Sun B. N. et al., 2018). The immune-enhancing effects of the polysaccharides in plant-based functional foods and their mechanisms are summarized in Table 1.

**TABLE 1:** Experiment and mechanism of polysaccharides bioactive components from plant-based functional foods on immune enhancement.

Source	Bioactive Compound	Model Type	Effective Dose	Effects	Mechanisms	References
*Dioscorea oppositifolia* L./Yam	yam polysaccharide	Lewis cells of lung cancer (B)	150 mg/kg	↑ IL-2, TNF-α, T lymphocyte proliferation, and the activity of NK cells.	They play an immune-enhancing role by promoting the activity of immune cells and related factors.	Xu et al. (2020a)
*Dioscorea oppositifolia* L./Yam	yam polysaccharide	Cyclophosphamide-induced immunosuppressive mice (A)	250 mg/kg	The liver index and white blood cell count significantly improved.	They achieve immune enhancement by regulating immune organs and cells.	Song et al. (2019)
*Codonopsis pilosula* (Franch.) Nannf./Codonopsis pilosula	codonopsis pilosula Polysaccharide	Cyclophosphamide-induced immunosuppressive mice (A)	200 mg/kg	It promotes lymphocyte proliferation *in vitro*. The thymus index and spleen index could be improved and the levels of IFN-γ, IL-2, IL-10, as well as serum IgG could be restored .	The thymus index, spleen index and immune related factors were improved, and the immunity function was enhanced.	Fu et al. (2018), Zou et al. (2019)
*Lycium chinense* Mill./Lycium chinense	lycium barbarum polysaccharide	Cyclophosphamide-induced immunosuppressive mice (A)	200 mg/kg	It enhances immune organ indexes and alleviating immune organ damage, enhance the production of immune-related cytokines (IL-2, IL-6, IL-1β, TNF-α and IFN-γ).	They play an immune-enhancing role by promoting the activity of immune cells and related factors.	Ding et al. (2019)
*Gynostemma pentaphyllum* (Thunb.) Makino/Gynostemma pentaphyllum	gynostemma pentaphyllum polysaccharide	Cyclophosphamide-induced immunosuppressive mice (A)	400 mg/kg	↑ IL-4, IgG, IgM, the spleen and thymus indexes, macrophage and splenic lymphocyte activity.	The thymus index, spleen index and immune related factors were enhanced.	Li et al. (2015), Bao et al. (2018)
*Ginkgo biloba* L*./*Ginkgo	ginkgobiloba leaves polysaccharide	SPF chickens (A)	40 g/L	↑ IL-2 and IFN-γ, CD4^+^ and CD8^+^ T lymphocyte number	Promote humoral immunity and cellular immunity, and have a good role in immune promotion	Meng et al. (2018b)
*Platycodon grandiflorus* (Jacq.) A. DC./Platycodon	platycodon polysaccharide	SPF mice cells (B)	100 μg/ml	It can selectively activates B cells and macrophages.	They play an immune-enhancing role by promoting the activity of immune cells.	Lv et al. (2016)
*Acanthopanax senticosus* (Rupr. Maxim.) Harms/Acanthopanax	angelica sinensis polysaccharide	Solid tumor mouse (A)	200 mg/kg	↑ immune organ index, TNF-α, IL-1β and IL-6	TLR4 signaling pathway may be involved in the immunomodulatory effects.	Zhou et al. (2017)
*Panax ginseng* C. A. Meyer/Ginseng	ginseng Leaf Polysaccharide	chickens (A)	20 g/kg	↑ the mmune organs index, IL-2, IL-12 TNF-α, the activity of NK cells.	It can promote the development of immune organs and secretion of related cytokines, so as to improve immune performance.	Wei et al. (2019), Hwang et al. (2018)
*Pseudostellaria heterophylla*? (Miq.) Pax Pseudostellariae Radix	radix Pseudostellariae polysaccharide	Cyclophosphamide-induced immunosuppressive mice (A)	50 mg/kg	↑ immune organ index, IgA, IgG, IgM, IL-2, IL-4, IL-6 and IFN-γ.	It can promote the development of immune organs and secretion of related cytokines.	Feng et al. (2020)
*Litchi chinensis* Sonn./Litchi	litchi pulp polysaccharides	Cyclophosphamide-induced immunosuppressive mice (A)	200 mg/kg	↑ IL-6, TNF-α, IgG, IgA, IgM, immune organ index.	It can stimulate the proliferation of splenocytes, balancing the ratio of spleen lymphocyte subsets, up-regulating the thymus and spleen indices.	Huang et al. (2016)
*Atractylodes macrocephala* Koidz./Atractylodes	*atractylodis macrocephalae* Koidz. polysaccharides	Mouse lymphocytes (B)	200 μg/ml	↑ IL-2, IL-6, IL-10 and the proliferation of T lymphocytes.**↓** TNF-α and IgG	It can promote lymphocyte proliferative response	Xu et al. (2020b)
*Ziziphus jujuba* Mill./Jujube	jujube Polysaccharide	Mouse lymphocytes (B)	160 μg/ml	↑ the mRNA of IL-2, IL-6, IL-10, IL-12 and lymphocyte multiplication	It can improve the immune function by inducing lymphocyte proliferation, lymphocyte cytokine secretion and mRNA expression.	Li et al. (2021)

Extracted mulberry (*Morus alba* L.) leaf polysaccharides (MLP) exhibit a notable potential for improving immunity. More specifically, MLP can markedly improve the transformation rate of splenic lymphocytes and cytokines and notably increase the thymus index (Xue et al., 2015). Recently, Chen et al. studied the immune enhancement effects of MLP *in vitro* and demonstrated that, at concentrations of 125 and 250 μg/ml MLP, spleen B and T lymphocyte proliferation was promoted compared with control treatments (*p* < 0.05). For the *in vivo* experiments, chickens immunized with the Newcastle disease vaccine, were orally administered with MLP (4 and 8 g/kg); MLP markedly improved the Newcastle disease-associated serum antibody titer and serum IgA concentrations in tracheal and jejunal wash fluids (*p* < 0.05) (Chen et al., 2019). Another study evaluated the effects of MLP on various immune functions, including serum immunoglobulins and cytokines, in addition to lymphocyte proliferation in weanling pigs. These reports indicated that the thymus and spleen indices in the MLP groups (0.6 and 1.2 g/kg) were noticeably greater (*p* < 0.05) than those in the control group. Moreover, MLP supplementation elevated the levels of the serum cytokines interleukin (IL)-1, IL-2, IL-6, IL-8, and interferon (IFN)-γ, and increased the lymphocyte transformation rate (Zhao X. J. et al., 2019). These results suggest that MLP can markedly enhance immunity, and therefore can be used as an immune-enhancing drug candidate.

*Astragalus* polysaccharide (APS) has been proven to be non-toxic in long-term clinical trials (Huang et al., 2019). More importantly, APS exhibits a wide range of pharmacological effects related to immune system regulation, such as enhancing the immune organ index, promoting the proliferation of immune cells, stimulating the release of cytokines, and affecting the secretion of immunoglobulins and the conduction of immune signals (Zheng et al., 2020). Chen et al. used mouse macrophages (RAW264.7 cells) to study the effects of APS (0.1, 0.5, and 1.0 mg/ml) on their morphology and immune function after lipopolysaccharide (LPS) stimulation. Compared with the control group, treatment with 1.0 mg/ml APS markedly inhibited changes in macrophage morphology and the proliferation capacity caused by LPS stimulation; the activity of macrophage acid phosphatase was also significantly increased (*p* < 0.05). Moreover, at different concentrations, APS significantly alleviated the decrease in alkaline phosphatase activity (*p* < 0.05) and significantly reduced levels of the LPS-induced pro-inflammatory cytokines IL⁃1β and tumor necrosis factor (TNF-α; *p* < 0.05). After treatment with 1.0 mg/ml APS, toll-like receptor (TLR) 4, myeloid differentiation primary response gene 88 (MyD88), and nuclear factor-κB (NF-κB) mRNA expression levels in the macrophages were significantly reduced (*p* < 0.05). Therefore, APS can be used to improve the morphology of macrophages, to restore cell proliferation, to reduce the secretion of pro-inflammatory factors, and to attenuate the immune stress response by regulating the expression of genes related to the TLR4 signaling pathway (Chen Z. et al., 2020). Additionally, one clinical trial investigated the effect of APS injection on the inflammatory cell count and the levels of related factors in 196 patients with bronchial asthma. It was found that an APS injection combined with conventional treatment effectively reduced the inflammatory cell count. After 2 weeks of treatment, levels of IL-6, IL-8, IL-13 IL-17, and TNF-α, and inflammatory cell counts, were significantly lower than those of the control group; additionally, CD3^+^, CD4^+^, and CD4^+^/CD8^+^ levels were significantly higher than the control group (*p* < 0.05). The incidence of adverse reactions (two patients with sore throat cases) was 2.04%, which was lower than that in the control group (Qiu et al., 2018).

Thus, polysaccharides can exert their immune activity by activating immune cells. Owing to the complex structures of polysaccharides, they are less soluble in water, which affects their biological activity. Structural modification of polysaccharides may enhance their biological activities, and aid in determining the relationship between their structure and immune function.

### Saponins

Saponins are a type of aglycone containing triterpenoids or steroids. They are widely found to possess health-promoting properties in functional foods (He et al., 2019). Extracted plant saponins have shown good performance in various biological studies, demonstrating anti-tumor and immune-enhancing regulatory properties (Gong et al., 2020). Pharmacological studies of a variety of saponin compounds have also shown that they exert immune enhancement effects (Rajput et al., 2007). As an example, the immunological enhancement of gypenosides, which are mainly distributed in *Gynostemma pentaphyllum* (Thunb.) Makino (Pang et al., 2017), was investigated for immunosuppression in mice. Previously, studies have shown that gypenosides can promote the development of rat immune organs as well as improve specific and non-specific immune functions (Ning et al., 2016). In a recent study, Dan et al. (2020) established a mouse model of immunodeficiency via induction with cyclophosphamide (80 mg/kg). Subsequently, gypenosides (60, 120, and 180 mg/kg) were provided orally for 30 days. Compared to the model group, the peripheral blood white blood cell count, organ index, and CD4^+^/CD8^+^ levels were significantly increased (*p* < 0.01). The effect was more evident in the high-dose gypenoside group. Additionally, gypenosides were found to markedly increase the expression levels of TNF-α, IFN-γ, IL-10, IL-6, and IL-2 in serum, and their mRNA expression in spleen lymphocytes. Thus, gypenosides can enhance the immune function in mice with immunosuppression induced by cyclophosphamide treatment.

*Codonopsis pilosula* (Franch.) Nannf. is recognized as a medicine food homology species (Jiang et al., 2016); furthermore, the total saponins of *C. pilosula* (TSCP) have been studied for their immune-enhancing effects in mice with immunosuppression induced by hydrocortisone treatment. Interestingly, oral administration of TSCP enhanced the immune function in a dose-dependent manner (50, 100, and 200 mg/kg) and significantly increased levels of serum IL-2 and IFN-γ, proliferation of spleen T cells, and the killing rate of natural killer (NK) cells (*p* < 0.05). This indicates that TSCP can antagonize the inhibitory effect of hydrocortisone on T cells and NK cells, thus enhancing the immune function in immunosuppressed mice. Furthermore, compared with the model group, TSCP significantly improved the half hemolysis value for serum hemolysin and the phagocytosis index in the immunosuppressed mice (*p* < 0.05) (Cao and Wang, 2019). The results indicate that TSCP can enhance the phagocytic activity of mononuclear macrophages and promote the production of antibodies to restore them to the normal level in immunosuppressed mice. These results indicate that TSCP can enhance immunomodulatory effects in terms of cellular immunity, humoral immunity, and non-specific immunity.

**FIGURE 2:**
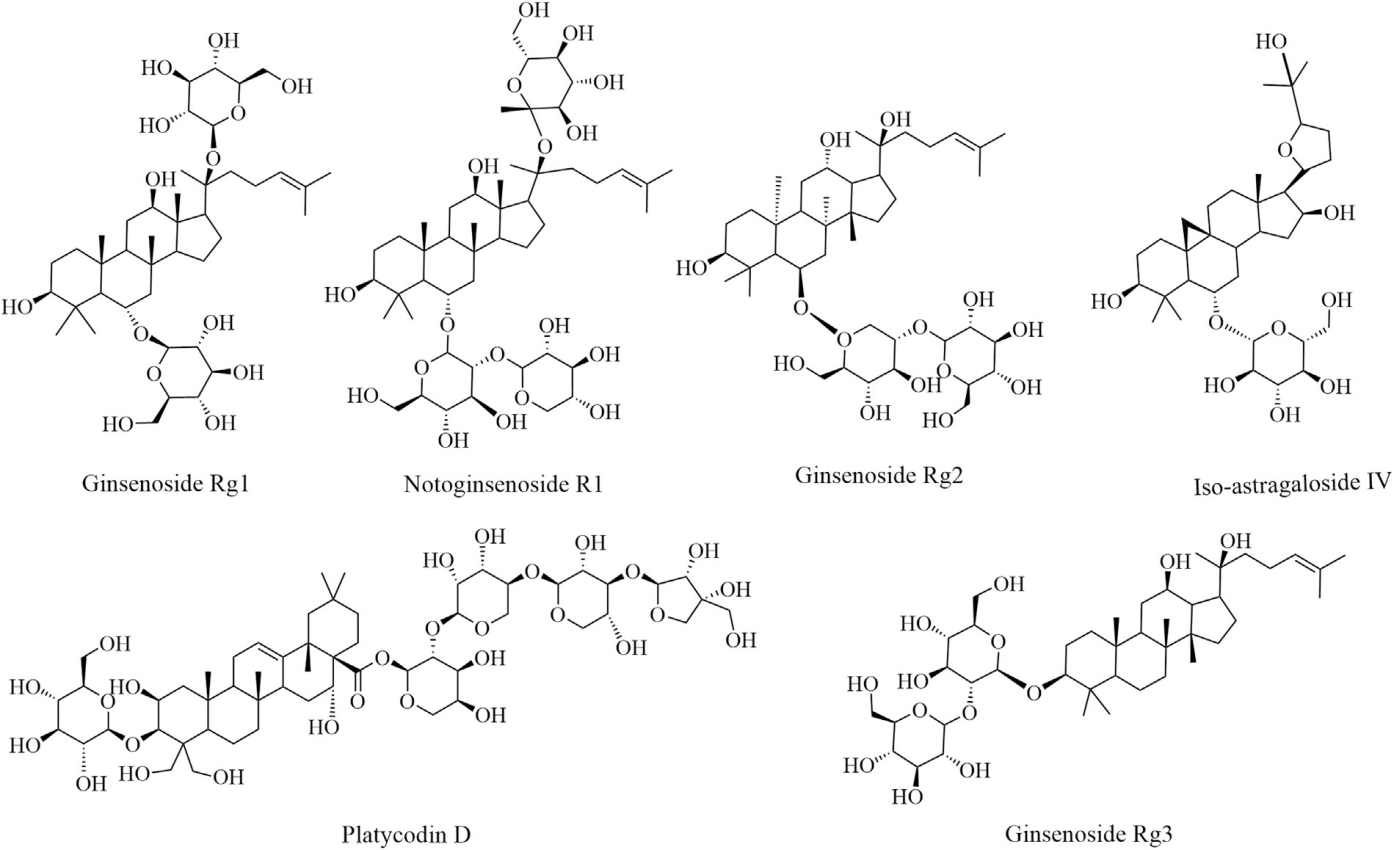
The immune enhancement effects of saponins in plant-based functional foods.

In summary, saponins can remarkably enhance immune function in various ways. However, saponins exhibit a hemolytic effect; therefore, the application dosage should be determined appropriately. Moreover, the mechanism of action of saponins relative to the signal pathway of the immune regulatory system warrants clarification. Additional research may help clarify the significance of saponins in food and drug development, which may provide a potential adjuvant for application in the future. The immune enhancement effects of the bioactive components of saponins and their structures are illustrated in Figure 2; experimental studies are summarized in Table 2.

**TABLE 2:** Experiment of saponins in plant-based functional foods on immune enhancement.

Source	Compound	Model Type	Effective Dose	Effects	Mechanisms	References
*Polygala tenuifolia* Willd/Polygala	polygala saponins	Cyclophosphamide-induced immunosuppressive mice (A)	400 mg/kg	↑ spleen index, thymus index, IL-2 ↓ IL-6	The mechanism is related to the regulation of cytokines.	Chai et al. (2018)
*Panax ginseng* C. A. Meyer/Ginseng	ginsenoside Rg1	Cyclophosphamide-induced immunosuppressive mice (A)	400 mg/kg	It could significantly increase the positive rate of antibody , spleen and bursa index and intestinal total sIgA and specific sIgA content compared with the C group, as well as up-regulated the mRNA expression of TLR4, p65, TGF-β, pIgR and CCR9genes in the chicken duodenum.	It acts on TLR4 receptors to exert immunomodulatory effects.	Bi et al. (2019)
*Panax notoginseng* (Burk.)F.H.Chen/Notoginseng	notoginsenoside R1	Traumatic shock rats (A)	50 mg/kg	↓ TNF-α, IL-1β, iNOS, p-p65, ERK1/2/p-ERK1/2 ↑ IL-10	It can enhance the immune response by blocking ERK1/2 and NF-κB signaling pathways, and regulate expression levels of inflammatory factors.	Wang et al. (2019b)
*Camellia oleifera* Abel./Camellia seed	tea saponin	Cyclophosphamide-induced chickens (A)	5 mg/kg	The lymphocyte proliferation and serum virus-specific antibodies were increased.	It could increase the specific antibody response to enhance immune function.	Chi et al. (2017)
*Panax ginseng* C. A. Meyer/Ginseng	ginseng stem-leaf saponins	Vaccinated mice (A)	50 μg	↑ IgG1, IgG2a, IgG2b, IFN-γ and IL-4 Increased splenocyte proliferative.	It could enhance cellular and humoral immune response to enhance immune function.	Xu et al. (2020c)
*Panax quinquefolium* L./American ginseng	american ginseng saponins	Rapamycin-induced zebrafish (A)	25 μg/ml	↑ IFN-γ Increased the number of neutrophils and macrophages.	It can increase the function of immune cells and promote the secretion of cytokines.	Lv et al. (2020)
*Panax ginseng* C. A. Meyer/Ginseng	ginsenoside Rg1	Cyclophosphamide-induced chickens (A)	1 mg/kg	↑ SIgA, TLR4, p65, TGF-β, pIgR, and spleen and bursa of Fabricius index	It can restore the function of suppressed immune organs to improve the immune response.	Bi et al. (2019)
*Panax ginseng* C. A. Meyer/Ginseng	ginsenoside Rg1	Type Ⅲ prostatitis rats (A)	40 mg/kg	**↓** IL?8, IL4, TNF-α, TGFβ	It can regulate immune balance by inducing the secretion of cytokines.	Sun et al. (2017)
*Panax ginseng* C. A. Meyer/Ginseng	ginsenoside Rg2	Patients with lung cancer (C)	0.5 g/d	↑ CD3^+^, CD4^+^, CD4^+^/CD8^+^ **↓** CD8^+^	It can regulate lymphocytes to protect immune function.	Ma and Bai (2019)
*Panax ginseng* C. A. Meyer/Ginseng	ginsenoside Rg3	Patients with gastric cancer (C)	40 mg/d	↑ CD3^+^, CD4^+^, CD4^+^/CD8^+^, T lymphocyte transformation rate **↓** CD8^+^	It can regulate lymphocytes to protect immune function.	Chen et al. (2017a)
*Panax ginseng* C. A. Meyer/Ginseng	ginsenoside Rg3	Nasopharyngeal carcinoma in patients (C)	100 mg/L	↑ CD4^+^, CD4^+^/CD8^+^, IgG, CD 80/86, IgM, IL-2 **↓** IL-6, CD8^+^ Promoted the proliferation of lymphocytes	It can signifi cantly enhance the immune function by promoting the proliferation of lymphocytes, and regulating immune factors.	Chen et al., 2017a.
*Momordica charantia* L./Bitter gourd	total momordicoside	Nephropathy of rats (A)	20 mg/kg	**↓** IL-2, IL-6, TGF-β1	It can inhibit the expression of TGF-β1, thereby reducing the serum IL-2 and IL-6.	Chen et al. (2017b)
*Panax notoginseng* (Burk.) F. H. Chen/Notoginseng	notoginsenosideS-6	Con A-Splenic lymphocyte of mice (B)	1 μg/ml	Increased T, and B lymphocyte proliferative	It can significantly promote the proliferation of T and B lymphocytes and enhance immune function.	Qin et al. (2017)
*Panax notoginseng* (Burk.) F. H. Chen/Pseudo-ginseng	notoginsenosideS-6	Con A-Splenic lymphocyte of mice (B)	25 μg/ml	↑ IL-2	As an activator, IL-2 induces T lymphocytes to express IL-2R, which in turn induces the production of IL-2 to play an immune role.	Qin et al. (2017)
*Platycodon grandiflorus* (Jacq.) A. DC./Platycodon	platycodin D	Lymphocyte and macrophage of mice (B)	50 μg/ml	↑ IL-2, IL-4, TNF-α, and IL-12 Enhanced lymphocyte proliferation and macrophage phagocytosis	It can enhance the activity of lymphocytes and macrophages to regulate the immune function.	Li et al. (2019)
*Astragalus mongholicus* Bunge/Radix astragali	Iso-astragaloside VI	Lymphocyte and macrophage of mice (B)	50 μg/ml	↑ IL-1, IL-2, IL-6, and IL-12 p70, the phagocytosis of macrophages	It is related to the enhancement of the secretion capacity of dendritic cells, and the promotion of non-specific immune phagocytosis and lymphocyte proliferation.	Ruan et al. (2021)

### Flavonoids

Flavonoids are an important group of bioactive secondary metabolites found widely in plants (Liu J. et al., 2018). They are also important natural bioactive ingredients in several plant-based functional foods (Jiang et al., 2020). In recent years, the immune-enhancing effects of flavonoids have attracted considerable attention. For example, soybean flavone, sea buckthorn flavone, and quercetin are known to exert good immune regulatory effects, and are highly effective natural immune enhancers, which can enhance cytotoxic T cells and the killing activities of NK cells, promote the release of cytokines, improve the immune organ index, enhance immune function, and promote the body's immune system (Rasouli and Jahanianet, 2015; Kamboh et al., 2016). Additionally, flavonoids can inhibit the production of pro-inflammatory cytokines by regulating NF-κB expression (Huang et al., 2018).

Hesperidin is a beneficial bioactive ingredient mainly found in citrus fruits (Tejada et al., 2018). A recent study investigated the effects of oral hesperidin on the systemic immune system of rats after completion of intense exercise regimens. Supplementation of hesperidin (200 mg/kg) significantly reduced the leukocytosis induced by intensive exercise, increased the cytotoxicity of NK cells, and increased the proportion of phagocytic monocytes and T helper cells in thymus, blood, and spleen. Additionally, cytokine (IL-6, IFN-γ) secretion in peritoneal macrophages decreased (*p* < 0.01) (Ruiz-Iglesias et al., 2020). Moreover, *in vitro* studies have shown that hesperidin can play an anti-inflammatory role by mediating immune-related pathways, namely the NF-κB pathway (Birsu Cincin et al., 2015).

*Lonicera japonica* is recognized as an edible and medicinal species (Zhang B. et al., 2019). In this context, *L*. *Japonica* flavone has been studied for its immunomodulation effects in mice with dexamethasone-induced immunosuppression. Compared with the model group, *L. japonica* flavone (400 mg/kg) significantly improved the organ indices (*p* < 0.01). This indicates that it exerts remarkable protective effects on the spleen and thymus. Moreover, the flavone significantly enhanced the activities of non-specific immune factors (alkaline and acid phosphatases) in serum. *L. japonica* flavone could significantly increase the content of superoxide dismutase, as well as reduce the activities of monoamine oxidase and malondialdehyde in spleen and thymus in immunosuppressed mice (*p* < 0.01) (Pi et al., 2015). Recently, a clinical trial was conducted to investigate the effect of honeysuckle soup combined with benzathine penicillin on serum IL-8 and TNF-α levels in patients with syphilis. The serum IL-8, and TNF-α levels were significantly lower than those in benzathine penicillin-treated patients (*p* < 0.05) (Ni and Zhu, 2020). Thus, this suggests that *L. japonica* extract exhibits good immunomodulatory effects.

**FIGURE 3:**
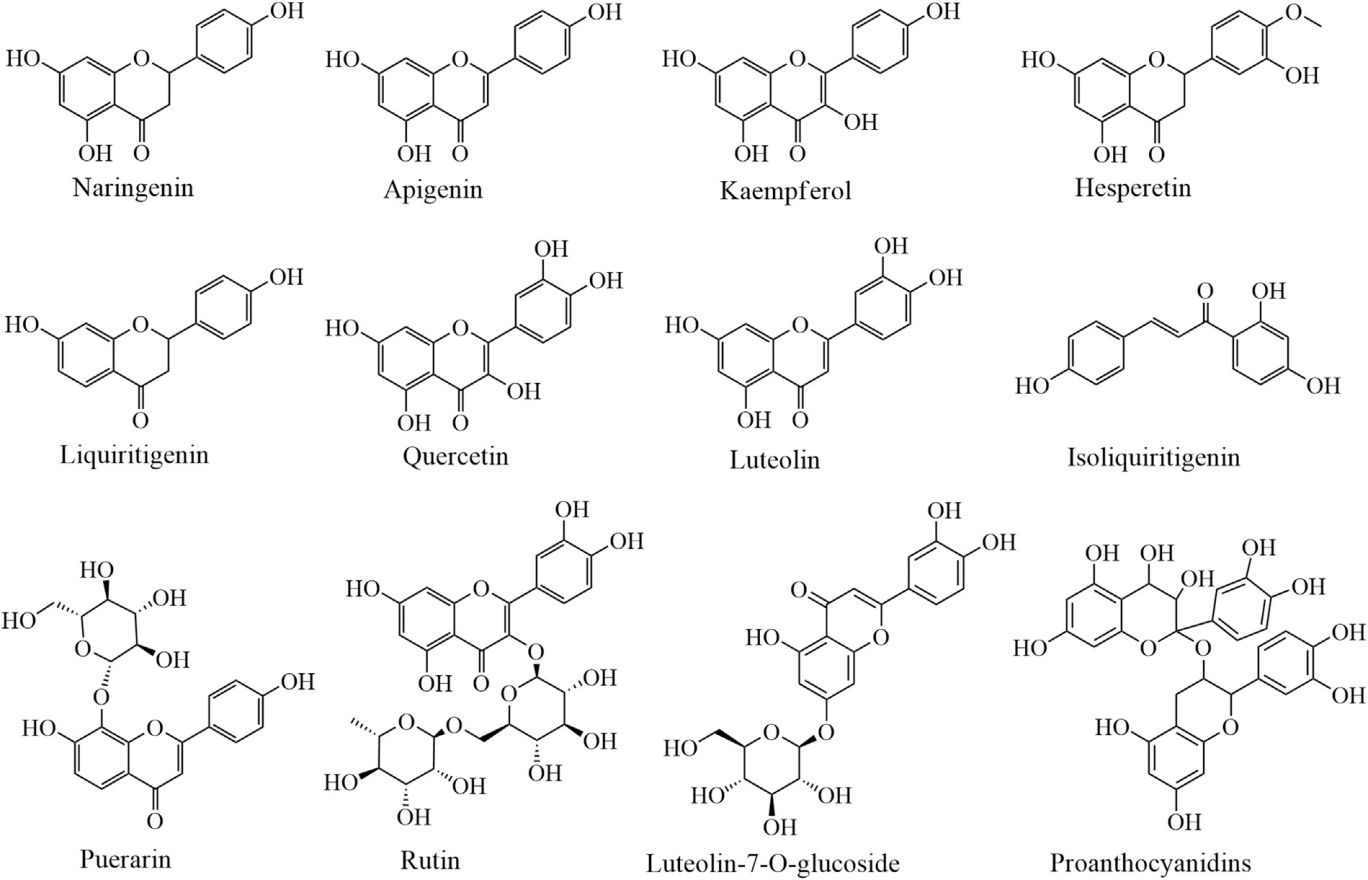
The immune enhancement effects of flavonoids in plant-based functional foods.

In recent years, the immune-enhancing effect and mechanism of action of flavonoids have been studied extensively; however, their clinical application as immunoregulatory drugs is limited because of their poor pharmacokinetic profile (De Ferrars et al., 2014). Furthermore, the large number of active sites, relatively slow efficacies, and the lack of specificity and selectivity towards certain diseases has limited the clinical application of flavonoids. It is believed that a gradual elucidation of the immune enhancement effect and mechanism of action of flavonoids will lay a foundation for the preliminary study of clinical immune enhancement applications. The immune enhancement effects of flavonoids from plant-based functional foods are summarized in Table 3; their structures are illustrated in Figure 3.

**TABLE 3:** Flavonoids components in plant-based functional foods on immune enhancement.

Source	Compound	Model Type	Effective Dose	Main Results	Mechanisms	References
*Apium graveolens* L./Celery	Apigenin	LPS-induced spleen cells (B)	20 μM	**↓** IL-1β, IL-6 and TNF-α. The expression of CD80, CD86 and MHCII of DCs were inhibited.	The mechanism is related to inhibiting DCs activation and function.	Liu et al. (2017c)
*Glycine max* (Linn.) Merr./Soybean	soy isoflavones	Rats (A)	50 mg/kg	**↓** IL-4, TNF-α, and INF-γ.	It can regulate the expression levels of cytokines to enhance immunity.	Li et al. (2017)
*Glycyrrhiza uralensis* Fisch./Licorice	isoliquiritigenin	LPS-induced bone marrow-derived macrophages (B)	40 μM	**↓** the protein levels of IL-1β, IL-6.	It may inhibit Mincle/Syk/NF-kappa B signaling pathway in macrophage to exert anti-inflammatory effects.	Liao et al. (2020)
*Ginkgo biloba* L./Gingko	proanthocyanidins	Cyclophosphamide-immunosuppression mice (A)	100 mg/kg	↑ Thymus and spleen index, TNF-α, and TNF-α mRNA. **↓** IL-10, and IL-10 mRNA.	It can regulate the secretion of cytokines and enhance immunity.	Kong et al. (2018)
*Ginkgo biloba* L./Gingko	proanthocyanidins	B16F10 tumor cells (B)	80 μg/ml	↑ IL-12, and TNF-α T cell-mediated immune responses increased.	It enhances the immune response mediated by T cells and plays an immunological enhancement role.	Zhang et al. (2017), Zhang et al. (2019b)
*Hippophae rhamnoides* L./Sea-buckthorn	seabuckthorn flavones	Dendritic cell (B)	200 μg/ml	↑ antigen presenting molecules HLA-DR, CD80, CD83 and CD86.	It can improve the antigen presentation ability of dendritic cell and promote the maturation of dendritic cell phenotype.	Liu et al. (2017c)
*Lonicera japonica* Thunb./Flos Lonicera japonica	luteolin	LPS-induced RAW264.7 cells (B)	1 μM	**↓** IL-6, TNF-α, NF-κB and phosphorylation of IκB.	It regulate NF-κB and the secretion of cytokines to enhance the immunoregulative effect.	Cheng and Yeh (2019)
*Lonicera japonica* Thunb./Flos Lonicera japonica	luteolin-7-O-glucoside	LPS-induced mice splenocytes, and macrophages (B)	4.48 μg/ml	NK cell activity and the proliferation of T lymphocytes increased, as well as macrophage lysosomal activity decreased.	It exhibites important immune enhancement activity by regulating NK cell activity and the proliferation of T lymphocytes.	Maatouk et al. (2017)
*Sophora japonica* L./Fructus Sophorae	genistein	LPS-induced broiler chicks (A)	5 mg/kg	The immune spleen, thymus and bursa indices were increased.	It can promote the development of immune organs to play immune function.	Kamboh et al. (2016)
*Sophora japonica* L./Fructus Sophorae	rutin	Cyclophosphamide-induced rats (A)	50 mg/kg	The phagocytic index, total leukocyte count, and serum immunoglobulin levels were in increased. **↓** TNF-α, and IL-6 ↑ p38-MAPK, NFκB, i-NOS and COX-2 in liver.	It can stimulate humoral and cellular responses to enhance the immunoregulative effect.	Nafees et al. (2015), Manzoni et al. (2020)
*Allium cepa* L./Onions	quercetin	Chickens (A)	4 g/kg	The indexes of spleen, bursa of Fabricius and thymus, and the phagocytic function of macrophages were improved. ↑ IL-4, and IL-12	It can promote the development of spleen, bursa and thymus, improve the phagocytosis of macrophages, and induce the secretion of cytokine to improve the immune function of the body.	Li. X. et al. (2020)
*Eucommia ulmoides* Oliv./Eucommia ulmoides	quercetin	ConA or LPS-stimulated lymphocytes (B)	20 μM	↑ IL-2 and IFN-γ in lymphocytes.	It can promote lymphocyte proliferation and cytokine secretion.	Wang et al., 2016.
*Eucommia ulmoides* Oliv./Eucommia ulmoides	flavone from Eucommia ulmoides Oliv.	ConA or LPS-stimulated lymphocytes (B)	20 μM	↑ IL-2 and IFN-γ in lymphocytes.	It can regulate the secretion of cytokines and enhance immunity.	Wang et al., 2016.
*Kaempferia galanga* L./Rhizoma kaempferiae	kaempferol	Cold-stress mice (A)	25 mg/kg	**↓** IL-9, IL-13 and CD8^+^ T cells ↑ CD4^+^, CD4^+^/CD8^+^ T cells, and IFN-γ	It boosts immunity by inhibiting the activation of pro-inflammatory factors.	Jia et al, (2019)
*Pueraria lobata* (Willd.) Ohwi/Pueraria	puerarin	Gouty arthritis mice (A)	100 mg/kg	**↓** IL-1, and TNF-α ↑ IL-10, and NO The number of neutrophils and lymphocytes was significantly reduced.	It may play an immune-enhancing role by influencing the expression level of inflammatory cytokines.	Zhang et al. (2020b)
*Pueraria lobata* (Willd.) Ohwi/Pueraria	puerarin	Viral myocarditis in children (C)	40 mg/ml	↑ CD3^+^, CD4^+^, and CD4^+^/CD8^+^ **↓** IL-6, IL-8, and TNF-α	It can reduce the level of inflammatory factors to improve its immune function.	Wang et al. (2020a)

### Alkaloids

**FIGURE 4:**
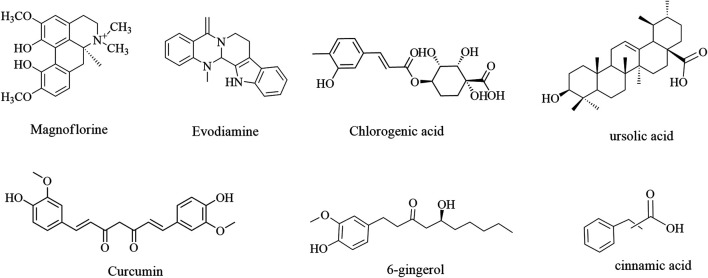
The immune enhancement effects of alkaloids and other components in plant-based functional food.

Alkaloids are nitrogenous compounds other than proteins, peptides, amino acids, and vitamin B that mainly exist in the plant kingdom (Song and Jiang, 2017). Owing to their complex structures and remarkable biological activities, their roles in immune enhancement should not be ignored (Jiang et al., 2020). Indeed, it is known that alkaloids play an immune-enhancing role by regulating the proliferation of thymic and splenic lymphocytes and the secretion of cytokine (Zhou et al., 2020). The immune enhancement of alkaloid structures is illustrated in Figure 4.

Magnoflorine has been isolated from *Magnolia officinalis* Rehd. et Wils and shown to activate the NF-κB and phosphatidylinositol 3-kinase-protein kinase B (PI3K-Akt) signaling pathways by promoting the expression of MyD88 and TLR4 to enhance the immune function of macrophages stimulated by LPS. The results showed that magnoflorine (50 μg/ml) can enhance the upregulation of TNF-α, IL-1β. Additionally, magnoflorine treatments augmented the phosphorylation of extracellular signal-regulated kinase (ERK), c-Jun N-terminal kinase (JNK), and p38 MAPKs (Haque et al., 2018). Moreover, magnoflorine can also promote the proliferation of spleen cells induced by LPS in rats, enhance the secretion of T_H_1 and T_H_2 cytokines, and elevate the number of CD4^+^ T cells (Ahmad et al., 2015).

Evodiamine is the main active component of *Euodia rutaecarpa* (Juss) Benth, and has been shown to regulate mouse immunity (Song et al., 2015). Evodiamine inhibits the proliferation of thymic and splenic lymphocytes induced by concanavalin A (ConA) in mice. More specifically, the results of ELISA tests showed that the release of IL-2 and IL-12 in the spleen and thymus cells of mice treated with evodiamine was significantly lower than that in the control group (*p* < 0.05). Furthermore, analysis of reverse transcription polymerase chain reaction results showed that the mRNA levels of Bcl-12 and CDK2 in cells treated with 0.75 μmol/L evodiamine were significantly higher than those in the control group. Compared to that of the control group, treatment with 0.75 μmol/L evodiamine induced apoptosis of the thymic and splenic cells in mice (*p* < 0.05) (Song et al., 2008).

Although alkaloids have been shown to play an immunomodulatory role by promoting or by suppressing the activation or differentiation of immune cells and by regulating cytokine expression, a lack of clinical data implies that further studies should be conducted in the context of clinical trials and toxic doses.

### Others

In addition to the above-mentioned bioactive ingredients, other components (e.g., terpenoids, essential oils, and organic acids) have also been found to exhibit immune enhancement effects. For example, 6-gingerol is the main component of ginger (*Zingiber officinale* Rosc.) The infiltration of CD4^+^/CD8^+^ T cells and B cells was increased, and the number of CD4^+^ T cells was decreased, in tumor-bearing mice after treatment with 6-gingerol (Fan et al., 2021). Moreover, the cinnamic acid present in cinnamon are known to elevate the levels of white blood cells (Cheng et al., 2017). The immune enhancement effects of others are summarized in Table 4; their structures are illustrated in Figure 4.

**TABLE 4:** Other components in plant-based functional foods on immune enhancement.

Source	Compound	Model Type	Effective Dose	Main Results	Mechanisms	References
*Curcuma longa* L./Turmeric	curcumin	Colitis mice (A)	200 mg/kg	**↓** IL-2, IL-12P40, IL-21, GM-CSF ↑ IL-4, IL-15, IL-23	It can enhance immunity by inhibiting the expression of pro-inflammatory factors and increasing the secretion of anti-inflammatory factors.	Wang et al. (2020c).
*Hippophae rhamnoides* L./Sea-buckthorn	ursolic acid	Mouse lymphocytes (A)	200 mg/kg	**↓** IL-2, ↑ IL-4, IL-15, IL-23	It can enhance immunity by inhibiting the expression of pro-inflammatory factors and increasing the secretion of anti-inflammatory factors.	Wang et al. (2020c).
*Lonicera japonica* Thunb./Flos Lonicera japonica	chlorogenic acid	LPS-induced RAW264.7 cells (B)	1 μM	**↓** IL-6, TNF-α, NF-κB	It regulate NF-κB and the secretion of cytokines to enhance the immunoregulative effect.	Cheng and Yeh (2019).

(A): *in vivo*.

(B): *in vitro*.

(C): in human.

↑: enhanced effects.

**↓**: inhibited effects.

## Mechanisms of the Immune-Enhancing Effects of Plant-Based Functional Foods

### Influence on Immune Signal Transduction

#### The TLR/NF-κB Signaling Pathway

**FIGURE 5:**
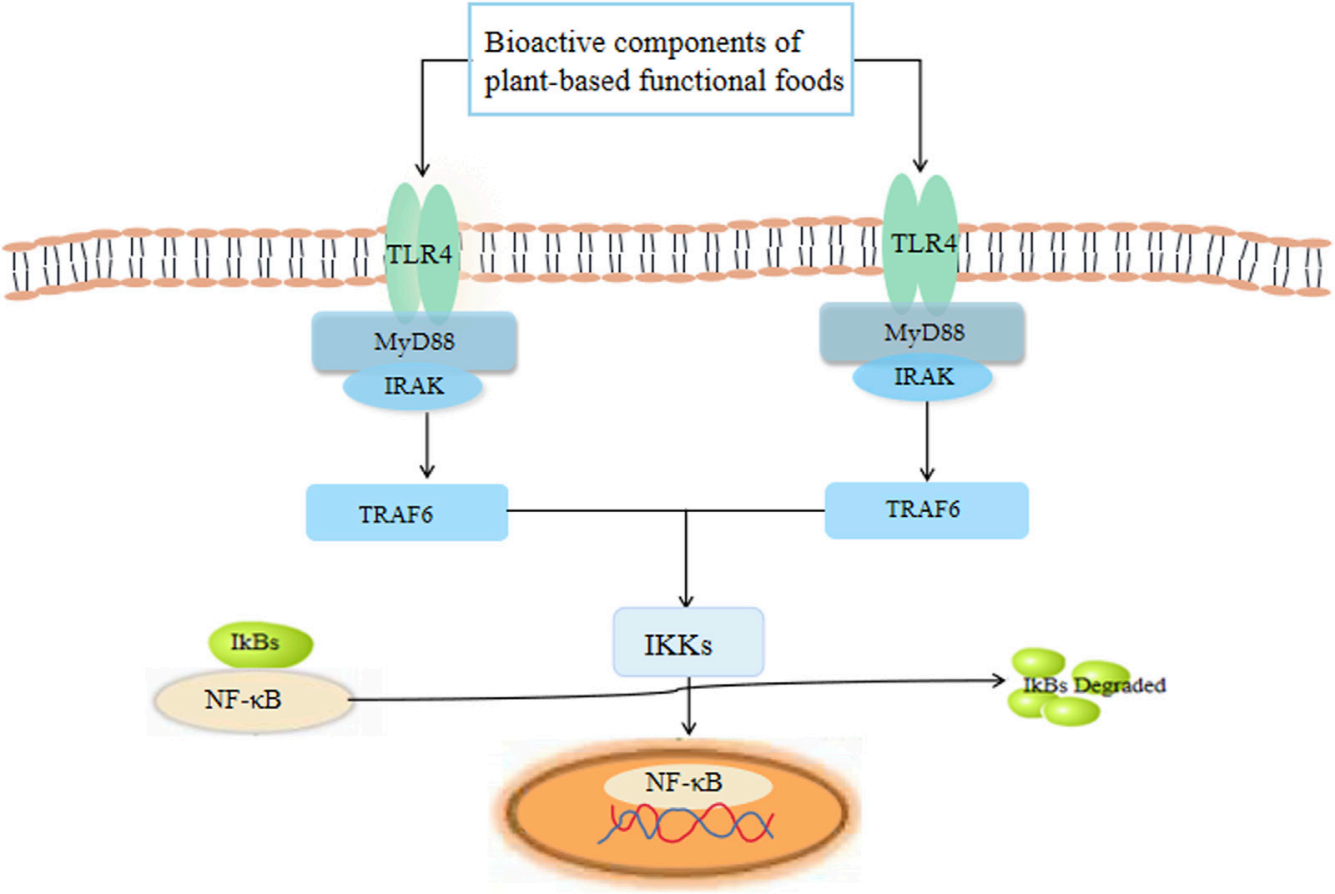
The receptors and signaling pathways triggered by plant-based functional foods.

NF-κB is one of the key factors that regulates cell gene transcription (Figure 5). The IκB kinase (IKK) complex is activated to catalyze the phosphorylation of IκB and to establish interaction with NF-κB. Activated NF-κB is transported to the nucleus, where it directly initiates and regulates the transcription of genes involved in the immune response, and regulates the expression of cytokines and adhesion molecules (Li and Verma, 2002). NF-κB signaling is critical for the expression of inflammatory cytokines. When cells are exposed to inflammatory stimuli, the stimuli act by regulating inflammation through the IKK-IκB-NF-κB inflammatory cytokine pathway (Yang et al., 2017). Therefore, NF-κB can regulate inflammatory mediators, cytokines, and adhesion molecules, affecting the innate or acquired immune response, inflammatory response, tumor growth, and other biological functions in the body (Wang et al., 2019). The appropriate intervention of NF-κB signaling after inflammation may be important to reduce further inflammation damage and the induction of other diseases (Hou et al., 2021). Importantly, recent studies have shown that bioactive substances from plant-based functional foods can exert an immunomodulatory effect by inhibiting NF-κB activation, and therefore migration (Yang et al., 2017).

TLRs are a class of innate immune-related pattern recognition receptors (PRRs). TLRs can initiate innate immunity immediately upon infection by identifying the pathogen, and by initiating acquired immunity through signal transduction, which plays an important defensive role against a variety of microbes (Fitzgerald and Kagan, 2020). TLR signal transduction pathways can be divided into MyD88-dependent and non-MyD88-dependent pathways according to the different receptor proteins involved in the signal transduction process. MyD88-dependent pathways can be mainly divided into two types, namely the TLR-MyD88/IRAK-mitogen-activated protein kinase (MAPK) pathway and the TLR-MyD88/IRAK-NF-κB pathway (Fitzgerald and Kagan, 2020). More specifically, MyD88 establishes interaction with IRAK to recruit downstream signaling molecules to induce the activation of NF-κB and activator protein-1, and to secrete pro-inflammatory cytokines to activate innate immune responses. The non-MyD88-dependent approach mainly involves TLR3 and TLR4, which play important roles in the immune response (Bahramabadi et al., 2019). TLR4 is mainly expressed in macrophages, although it also plays a role in the recognition of LPS and certain endogenous heat shock proteins (Yang et al., 2017).

Studies have also shown that TLRs can be stimulated by components present in plant-based functional foods; NF-κB in the nucleus is activated, promoting cytokine secretion and up-regulating the expression of co-stimulating molecules, thereby playing an immune-enhancing role. For example, APS can confer protection against sepsis-induced cardiac dysfunction by inhibiting expression of the TLR4/NF-κB pathway (Xu et al., 2019).

#### MAPK Signaling Pathways

MAPKs are a group of threonine-serine protein kinases, which can respond to a wide range of extracellular stimuli; they constitute one of the most important signal transduction systems (Gong and Jiang, 2003). Under external stimulation, MAPK is activated via double site phosphorylation, promoting phosphorylation of transcription factors that enter the nucleus and regulating transcription of related genes (Wang J. T. et al., 2019). Three main MAPK family members are known, namely ERK, JNK, and p38MAPK, which play important roles in the regulation of cell proliferation, inflammation, apoptosis, and other signal transduction pathways (Cai et al., 2020). Dong et al. showed that hesperidin could selectively regulate the MAPK pathway to influence the cellular immune response. More specifically, hesperidin upregulates the expression and activation levels of p38MAPK and JNK, thereby enhancing cellular autonomic immunity (Dong et al., 2014). Additionally, sea buckthorn flavonoids can also inhibit the phosphorylation of p38 and the MAPK pathway of stress-activated protein kinase/JNK, thus reducing the immune inflammatory effect (Jiang et al., 2017). Licorice was shown to inhibit the expression of p38MAPK, ERK1/2, and JNK, and alleviated the immune inflammatory response in mice with myocardial fibrosis (Zhang Y. et al., 2016). These results suggest that active ingredients can play immune-enhancing roles by decreasing the phosphorylation levels of ERK, JNK, and p38MAPK.

### Promotion of Immune Cytokine Production

Cytokines are small, low-molecular-weight proteins that have both autocrine and paracrine functions in immune cells, and are responsible for the regulation of the immune function of the body and participation in certain inflammatory reactions (Conlon et al., 2019). Immune cytokines not only affect the immune and hematopoietic systems, but also extensively affect the nervous, endocrine, and cardiovascular systems (McComb et al., 2019). Cytokines are classified according to their different functions, namely ILs, chemokines, lymphokines, IFNs, and the TNF family. As the most extensively secreted cytokine, ILs play an important role in the regulation of intercellular immunity and inflammation (McComb et al., 2019). Rapid advances in the therapeutic use of IL-2 and IL-10 against cancer have recently been achieved (Qiao and Fu, 2020). IFN is a subset of cytokines; they possess anti-viral, anti-tumor, and immune regulation abilities (McComb et al., 2019). The TNF family can be divided into two types, namely TNF-α and TNF-β, which can participate in immune regulation and inflammation (McComb et al., 2019). Among them, TNF-α is a class of dual action cytokines that play a dominant role in the regulation of inflammation (Wang et al., 2020d). Normally, cytokines play an important role in the maintenance of body homeostasis by regulating the body's immune response at a low level by controlling the development, differentiation, and function of immune cells (Zhang L. L. et al., 2020). On the contrary, a variety of pro-inflammatory cytokines are rapidly and considerably produced in body fluids, which can affect cell function, intracellular signaling pathways, and diseases (Du et al., 2021). Presently, plant-based functional foods are known to enhance immunity by promoting the production of cytokines. However, certain components in functional foods can inhibit the increase in cytokines caused by the inflammatory response, thereby protecting cells in the body (Zheng et al., 2020).

Wang et al. used RAW264.7 macrophages to study the anti-inflammatory and immunomodulatory effects of TFA. After a treatment period of 12 h, 10 μg/ml TFA significantly increased the secretion of IL-1β, IL-6, and TNF-α, and the mRNA levels of IL-1β, IL-6, and TNF-α in normal RAW264.7 cells. However, TFA (10, 25, and 100 μg/ml) inhibited the overexpression of IL-1β, IL-6, and TNF-α and their enhanced transcription levels in LPS-stimulated RAW2.7 cells in a dose-dependent manner. TFA enhances cytokine and mRNA levels under normal conditions, and inhibits the excessive release of proinflammatory cytokines and mRNAs under the stimulation of LPS, thus exerting its anti-inflammatory and immunological bimodulation effect (Wang M. et al., 2020).

### Promotion of Innate Immunity Function

Innate immunity comprises a series of defense mechanisms that play crucial roles in the initiation and action of specific immunity. Thus far, various studies have found that the regulation of innate immune cells, such as NK cells, DCs, and macrophages, plays an immune-enhancing role (Liu C. H. et al., 2017).

#### Effects on NK Cells

NK cells are a type of immune cell closely related to responses during tumor formation, virus-associated infections, and immune regulation; they are the first line of defense in the human body (Russick et al., 2020). NK cells express several activation and inhibitory receptors, and secrete cytokines and chemokines to enable interaction with other immune cells (Vivier et al., 2018). Additionally, NK cells play a key role in tumor immune surveillance, generating a coordinated anti-tumor immune response through their cytotoxic effect function and their ability to interact with other immune cells (Morsink et al., 2020). Regulation of the activity of NK cells can strengthen the immune system against diseases.

Maatouk et al. investigated the ability of naringenin (5, 10, and 21 μg/ml) and heated naringenin (4, 6, and 8 μg/ml) to enhance NK activity against K562 myelogenous leukemia cells. Their findings revealed that naringenin and heated naringenin improved the NK cell lysis activity at concentrations of 5 and 6 μg/ml, respectively, whereby for heated naringenin, this enhancement was dose-dependent. Notably, naringenin treatment resulted in an enhanced NK cytotoxic activity against these target cells (Maatouk et al., 2016). Furthermore, Valentová et al. (2016) showed that rutin exposure also increased the killing activity of NK cells; rutin is commonly found in the normal human diet and is increasingly used in food supplements.

#### Effects on DCs

DCs are the most effective antigen-presenting cells in the innate immune system that play a vital role in both immune homeostasis and antitumor activity. As key immune sentinels, these cells initiate and regulate adaptive immune responses by integrating and transmitting a substantial number of afferent signals to lymphocytes (Ding et al., 2018). Additionally, a myriad bioactive components play immunomodulatory roles by promoting the development and maturation of DCs. Therefore, modulation of the DC activity to ameliorate autoimmune diseases may be effective (Lin et al., 2017; Lin et al., 2020).

Proanthocyanidins are found in dietary components (Kong et al., 2018). Williams et al. (2017a) suggested that proanthocyanidins could induce the formation of an anti-inflammatory phenotype in human DCs, resulting in the selective downregulation of the T_H_1 response in naive T cells. Furthermore, Zhang et al. (2015) showed that chrysin could inhibit the functional differentiation and maturation of DCs and improve the inflammatory response in experimental autoimmune encephalomyelitis.

#### Effects on the Mononuclear Phagocyte System (MPS)

The MPS consists of immune effector cells distributed in blood, lymph, and tissues. Macrophages are key host defenses against pathogens and play an important role in the immune system, including antigen presentation, phagocytosis of pathogens, secretion of various cytokines, and activation of immune responses (Tao et al., 2020). Macrophages can phagocytize pathogens directly, but also indirectly attack pathogens through the release of cytotoxic molecules such as nitric oxide (NO) and secretion of cytokines, including TNF-α and IL-6, to perform immune functions (Zhang X. X. et al., 2016; Dong et al., 2019). Biologically active ingredients derived from plant-based functional foods can activate macrophages, enhance phagocytosis and antigen presentation, and promote the secretion of relevant active molecules (Lv et al., 2016). Thus, macrophages are ideal target cells for a variety of immunomodulatory and anti-inflammatory drugs.

Ginseng polysaccharide (GPS) is extracted from *Panax ginseng* C. A. Meyer (Zhao B. et al., 2019). In one study, the effects of GPS on the morphology and immune function of LPS-induced mouse macrophages (RAW264.7) were determined. The results showed that GPS significantly improved macrophage morphology and restored proliferation. Compared with the LPS group, treatment with 1 mg/ml GPS significantly increased macrophage acid phosphatase activity, while treatments with 0.5 and 1 mg/ml GPS significantly reduced alkaline phosphatase activity caused by LPS stimulation (Chen G. Y. et al., 2020). The active components of *Platycodon grandiflorus* are mainly saponins, among which platycodin D is the main saponin constituent (Ji et al., 2020). Li et al. (2019) explored the effect of platycodin D on the immune function of mouse macrophage phagocytosis using an MTT assay. Compared with the control group, platycodin D (25, 50, 75, and 100 μg/ml) treatment significantly promoted the proliferation of lymphocytes and enhanced macrophage phagocytosis (*p* < 0.01). The group treated with 50 μg/ml platycodin D exhibited a more significant effect than that of the other groups (*p* < 0.01). Furthermore, platycodin D treatment stimulated the secretion of TNF-α and IL-12 in macrophages, with 50 μg/ml being the optimal concentration. Overall, it was found that platycodin D enhanced the activity of mouse macrophages.

### Enhanced Adaptive Immunity

Initial immune cells exhibit broad specificity against pathogens, whereas the adaptive immune system cells exhibit a highly specific immune response to a specific antigen (Wang et al., 2020d).

#### Enhanced Cellular Immunity

Cellular immunity is the immune response mediated by T lymphocytes (Nicholson, 2016). In cellular immunity, antigens are processed by antigen-presenting cells into peptides that bind to the major histocompatibility complex (MHC) and produce an activated T-cell receptor signal. The antigen binds to relevant receptors on the surface of T lymphocytes to produce a signal, and the T lymphocytes proliferate and differentiate rapidly with a few of them becoming sensitized lymphocytes. Among them, cytotoxic T cells (Tc) can lead to the rupture and death of exogenous cells, and T helper (Th) cells secrete cytokines such as ILs, which stimulate Tc and various phagocytes to accumulate around foreign cells and result in complete removal. Toward the end of this response, T suppressor (Ts) cells exhibit functions and halt the immune response by inhibiting the action of other lymphocytes (Nicholson, 2016; Wang and Lin, 2019). A series of studies have shown that plant-based functional foods can promote lymphocyte transformation and improve cellular immune function. (Yang et al., 2017).

As an example, lentinan has been shown to increase macrophage toxicity to metastatic tumors by regulating the function of immune cells such as T lymphocytes and macrophages at multiple levels (Ahn et al., 2017). More specifically, lentinan can promote T lymphocyte proliferation and enhance the T lymphocyte activity to improve the host body balance. Additionally, lentinan has been shown to stimulate T cells and improve survival in cancer patients (Wang J. T. et al., 2019).

#### Enhanced Humoral Immunity

Humoral immunity is mainly mediated by B cells, that is, plasma cells produce antibodies to protect the immune mechanism. The antibody titer reflects the affinity and immune response of the antibody to the antigen. The content of hemolysin in serum is an important indicator of humoral immune function (Xing et al., 2020). In this context, the active components of plant-based functional foods have been found to confer protection to the body by producing antibodies to accelerate lymphocytic phagocytosis and clearance by phagocytes (Yang et al., 2017).

The effect of polysaccharide extracted from the herb *Gastrodia elata* on humoral immunity in immunodeficient mice was studied following induction using cyclophosphamide (Li et al., 2016). After completion of a 10 days treatment, *G. elata* polysaccharide medium-dose (200 mg/kg) and high-dose groups (400 mg/kg) exhibited significantly increased serum IgA, IgG, and hemolysin levels (*p* < 0.01). Furthermore, the spleen index and thymus index in the high-dose polysaccharide group were increased, and serum IgM levels in the medium-dose polysaccharide group were significantly increased (*p* < 0.05). *G. elata* polysaccharide can alleviate the inhibitory effect of cyclophosphamide on humoral immune function in mice.

### Promotion Effect on Immune Organs Function

**FIGURE 6:**
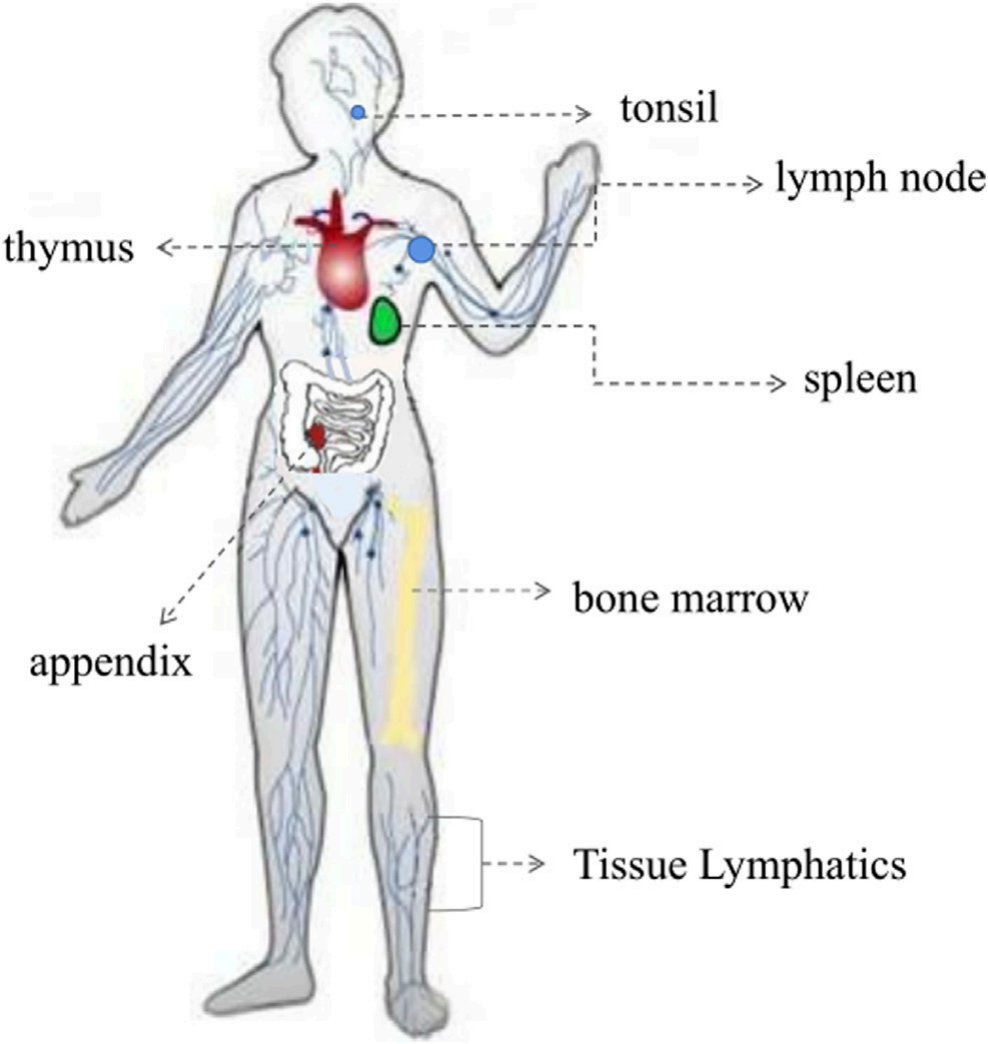
The immune system of immune organs.

The immune system is a defense network encompassing the entire body. Immune function is dependent on the immune organ to produce a considerable number of immune cells to regulate the immune response and to prevent the spread of infection (Figure 6; Yang et al., 2017; McComb et al., 2019). All blood cells originate from the same precursor hematopoietic stem cells, but their sites of maturation and residence differ, and the cells are mainly divided between the central immune organ and the peripheral immune organ (McComb et al., 2019). The thymus, as the primary immune organ, is mainly involved in the cellular immune response, whereas the spleen, as a peripheral immune organ, is involved in humoral immunity (Nicholson, 2016; Sun T. T. et al., 2018). Thus far, several studies have shown that components of plant-based functional foods can act on various immune organs, enhancing the immune response by increasing the organ weight, by improving the organ index (a reflection of a change in body immune function), and by promoting the development of partial visceral organs (Li X. Q. et al., 2020).

For example, *Lycium barbarum* polysaccharides (LBP) exhibit an enhancing effect on the immune organ index, which has been confirmed in a few studies (Hao et al., 2015; Zhao et al., 2015). Furthermore, Tang and He (2013) evaluated the effects of LBP on a D-galactose aging mouse model and found that the thymus index and spleen index were significantly increased in the LBP (3 g/kg) groups compared to those of the control (*p* < 0.01). Therefore, LBP can effectively protect the immune organs and enhance the immune ability of the body. Furthermore, Kamboh et al. (2016) investigated the immunomodulatory effects of hesperidin on LPS-induced broilers and showed that 20 mg/kg hesperidin treatment significantly increased the bursa and spleen index at 21 and 42 days compared to the control (*p* < 0.05). These results indicate that the bioactive components present in plant-based functional foods promote the growth and development of the immune organs and exhibit immune-enhancing effects.

## Conclusion

Immune dysfunction in the body can be caused by a variety of factors. To address this issue, plant-based functional foods have received increased attention because of their extensive immune-enhancing properties. As a result, various studies have attempted to elucidate the cellular and molecular regulatory mechanisms and signaling pathways of immunoactive ingredients to determine the immune-enhancing effects of these active ingredients. Unlike current drugs, which are expensive and can result in a variety of side effects, plant-based functional foods result in fewer side effects, are stable, and tend to exhibit a lasting efficacy. With the discovery and utilization of functional foods, suitable candidates among the vast range of natural products are being identified and characterized in detail. However, the application of bioactive compounds as immune factors has certain limitations; thus, we herein propose the following points and recommendations: 1) Although certain plant-based functional foods have been reported to exhibit beneficial effects in enhancing immunity, their bioactive components have not been fully elucidated and identified; hence, this should serve as a major focus for further investigations. 2) Presently, it is difficult to correlate the structures and activities of complex bioactive constituents (e.g., polysaccharides); thus, a study of the relationship between the structures and efficacies of bioactive components from plant-based functional foods is necessary. 3) Various active ingredients derived from plant-based functional foods remain to be tested in clinical trials. In this context, it should be noted that the effects observed in animal models may differ from those in humans, rendering human clinical trials essential. 4) The *in vivo* environment is more complex than the *in vitro* environment; thus, additional *in vivo* studies should be performed to further elucidate the mechanisms underlying the immune-enhancing capabilities of bioactive components obtained from plant-based functional foods. The molecular mechanisms underlying the action of such compounds should be studied intensively. 5) A few reports on the immune enhancement effects of bioactive components from plant-based functional foods originate from poor-quality research attributed to inadequate methods. Therefore, high-quality studies of such bioactive components are warranted to establish their efficacy and to provide a more convincing theoretical basis for the synthesis of novel immune-boosting drugs.

## Abbreviations

APS, *Astragalus* polysaccharide; GPS, ginseng polysaccharide; LBP, *Lycium barbarum* polysaccharides; TSCP, total saponins of *Codonopsis pilosula*; MLP, mulberry leaf polysaccharides; LPS, lipopolysaccharide; ConA, concanavalin A; NO, nitric oxide; CD3, cluster of differentiation 3; DCs, dendritic cells; NK, natural killer; IL, interleukin; INF, interferon; TNF, tumor necrosis factor; TLR, toll-like receptor; ERK, extracellular signal-regulated kinase; IKK, IκB kinase; JNK, c-Jun N-terminal kinase; MAPK, mitogen-activated protein kinase; MPS, mononuclear phagocyte system; NF-κB, nuclear factor-κB; PRRs, pattern recognition receptors; MyD88, myeloid differentiation primary response gene 88; PI3K-Akt, phosphatidylinositol 3-kinase-protein kinase B
